# Retraction: Berberine Improves Kidney Function in Diabetic Mice via AMPK Activation

**DOI:** 10.1371/journal.pone.0190562

**Published:** 2017-12-28

**Authors:** The PLOS ONE Editors

Concerns have been raised that images presented in Figures 1 and 2 of this *PLOS ONE* article [1] are similar to images in other published works. Several of the figure legends and labels indicate that the images showing similarities represent different experiments.

Figure 2A AMPK Western blot in [1] is similar to the following:

- Figure 6b GAPDH panel in [2]
- Figure 5B GAPDH panel in [3]
- Figure 6B GAPDH panel in [4]
- Figure 1d AMPK panel in [5]
- Figure 1E AMPK panel in [6]
- Figure 3A GAPDH panel in [7]
- Figure 2B AMPK panels in [8]
- Figure 6C AMPK panel in [8]

Figure 2A P-AMPK Western blot in [1] is similar to the following:

- Figure 1E P-AMPK panel in [6]
- Figure 3A p27 kip1 panels in [7]
- Figure 2A P-AMPK panel in [8]

Figure 2A LKB1 Western blot in [1] is similar to the following:

- Figure 5C Occludin panel in [3]
- Figure 3a iNOS panel in [5]
- Figure 2A LKB1 panels in [8]

Figures 1A, 1B and 1C, including Western blots and charts, appear similar in [1] and [8].

Figure 2B P-AMPK and AMPK Western blots in [1] are similar to Figure 2B P-AMPK and AMPK panels, respectively, in [8].

Additionally, there is duplication of some text between the Results section of the *PLOS ONE* article [1] and the Results sections of [5–8].

The authors have stated that the Western blots for this article were performed by an external company. Following evaluation of the issues identified and the information provided by the authors, the concerns about the data reported in the article remain. In light of the concerns about the integrity of the data and text overlap, the *PLOS ONE* Editors retract this article. The corresponding author regrets this situation and extends his apology to the scientific community.

We notified the authors’ institution and all co-authors of this retraction. GJG, LZ agreed with the retraction. LNS, HBN, XLW did not respond.

## References

[1] Zhao L, Sun L-N, Nie H-B, Wang X-L, Guan G-J (2014) Berberine Improves Kidney Function in Diabetic Mice via AMPK Activation. PLoS ONE 9(11): e113398 https://doi.org/10.1371/journal.pone.0113398 25409232 10.1371/journal.pone.0113398 PMC4237447

[2] Yu H-Y, Cai Y-B, Liu Z. Activation of AMPK improves lipopolysaccharide-induced dysfunction of the blood–brain barrier in mice. Brain Injury. 2015;29: 777–784. doi: 10.3109/02699052.2015.1004746 25794165 10.3109/02699052.2015.1004746

[3] Zhao Z, Hu J, Gao X, Liang H, Liu Z. Activation of AMPK attenuates lipopolysaccharide-impaired integrity and function of blood–brain barrier in human brain microvascular endothelial cells. Experimental and Molecular Pathology. 2014;97: 386–392. doi: 10.1016/j.yexmp.2014.09.006 25220346 10.1016/j.yexmp.2014.09.006

[4] Hu M, Liu B. Resveratrol attenuates lipopolysaccharide-induced dysfunction of blood-brain barrier in endothelial cells via AMPK activation. The Korean Journal of Physiology & Pharmacology. 2016;20: 325–332. doi: 10.4196/kjpp.2016.20.4.325 Retraction in: Hu M, Liu B. The Korean Journal of Physiology & Pharmacology. 2017;21(2): 277. 27382348 10.4196/kjpp.2016.20.4.325 PMC4930900

[5] Yang Y, Zhao Z, Liu Y, Kang X, Zhang H, Meng M. Suppression of oxidative stress and improvement of liver functions in mice by ursolic acid via LKB1-AMP-activated protein kinase signaling. Journal of Gastroenterology and Hepatology. 2015;30: 609–618. doi: 10.1111/jgh.12723 25168399 10.1111/jgh.12723

[6] Jiang Y, Lei J, Feng F, Liang Q, Wang F. Probucol suppresses human glioma cell proliferation *in vitro* via ROS production and LKB1-AMPK activation. Acta Pharmacologica Sinica. 2014;35: 1556–1565. doi: 10.1038/aps.2014.88 25399650 10.1038/aps.2014.88 PMC4261125

[7] Fan G-H, Wang Z-M, Yang X, Xu L-P, Qin Q, Zhang C, et al Resveratrol Inhibits Oesophageal Adenocarcinoma Cell Proliferation via AMP-activated Protein Kinase Signaling. Asian Pacific Journal of Cancer Prevention. 2014;15: 677–682. doi: 10.7314/APJCP.2014.15.2.677 Retraction in: Asian Pacific Journal of Cancer Prevention. 2017;18(10): 2895. 24568477 10.7314/apjcp.2014.15.2.677

[8] Hu M, Liu B. Resveratrol via activation of LKB1-AMPK signaling suppresses oxidative stress to prevent endothelial dysfunction in diabetic mice. Clinical and Experimental Hypertension. 2016;38: 381–387. doi: 10.3109/10641963.2015.1131288 27149559 10.3109/10641963.2015.1131288

